# The transfer of ^137^Cs and heavy metals to tissues within the organs of snails

**DOI:** 10.1038/s41598-023-42580-6

**Published:** 2023-09-21

**Authors:** Zhanat Baigazinov, Sergey Lukashenko, Batiyash Silybayeva, Klara Zharykbasova, Zhanylkhan Bukabayeva, Nurlan Muhamediarov, Bagdat Kantbayeva, Balzhan Kozhakhmetova, Tuvshinsaikhan Ganbaatar, Edit Toth-Bodrogi, Miklos Hegedus, Tibor Kovacs

**Affiliations:** 1 Alikhan Bokeikhan University, Semey, Kazakhstan; 2 Institute of Radiochemistry and Radioecology, University of Pannonia, Veszprém, Hungary; 3 All-Russian Scientific Institute of Radiology and Agroecology, Obninsk, Russia; 4 Institute of Radiation Safety and Ecology, NNC RK, Kurchatov, Kazakhstan

**Keywords:** Environmental chemistry, Environmental impact, Ecology, Risk factors

## Abstract

This paper describes the dynamics of ^137^Cs accumulation and its concentration ratio as well as that of some stable elements in the body, shell, gastrointestinal tract and albumin gland of a particular species of snail (terrestrial gastropod), namely the Giant African snail (*Lissachatina fulica*), after the long-term ingestion of contaminated forage and/or soil. The activity concentration of ^137^Cs in the their bodies increased over the first 40 days of the experiment, after which the increase in the activity of this radionuclide significantly reduced. The distribution of ^137^Cs in the body of a snail decreases as follows: gastrointestinal tract ˃ body = albumin gland ˃ shell. It was found that the contribution of soil towards the contamination of their bodies by ^137^Cs is far less than that of forage. Although the biological availability of Pb and U in forage is one order of magnitude higher than in soil, the main contribution to the contamination of snails originates from soil.

## Introduction

One of the main problems with studying the transfer parameters of radionuclides and heavy metals into the organs of and products originating from animals, both wild and livestock, is the extensive range of transfer data, e.g. concentration ratios and transfer factors^1–5^. In some cases, the difference between them can be as great as four orders of magnitude^6^. Even though one of the main factors behind this could be the source of intake such as water, soil or forage, other parameters—e.g. the duration of ingestion, radionuclide species in the soil, composition of macro and trace elements in their diet, characteristic features of animals like their age and productivity, etc.—might also be significant. Studying the contribution of each factor and uncertainties in the evaluation of concentration ratios or transfer factors could solve the problem concerning the wide range of data points available, which is crucial in predictive modeling and biomonitoring.

The contribution and importance of unintentionally ingested soil contaminated with radionuclides have previously been addressed in studies by N. Beresford^7^. The bioavailability of radionuclides in various sources of intake was deemed the main factor determining the transfer of radionuclides into food consumed by ruminants^8^. In this regard, studying the contribution of individual components of the environment such as the soil and forage towards the radionuclide contamination of products is relevant. Furthermore, in some cases, it can be assumed that some radionuclides that are ingested in various ways, that is, from the air, water, forage or soil, can accumulate in organs, resulting in a possible additive effect.

In some cases, the ingestion of snails can be an important pathway for the intake of radionuclides and heavy metals as well as contribute to the internal radiation exposure^9,10^. In other cases, snails can be used to biomonitor various contaminants, including heavy metals and radionuclides^11,12^. Gastropods, as a reference organism^13–15^, are a relatively convenient object of study for assessing the transition of radionuclides in the chains of "soil to organisms" and "food to organisms"^16–18^. Studying the additive nature of the accumulation of radionuclides in the body through various pathways can necessitate significant revision of the approaches and methods of radioecological assessment of the environment; of the quality of animal products; as well as of their contribution to internal radiation exposure. The importance of soil adhered to vegetation has been described by Beresford and Howard for vertebrates^7^, which was also confirmed in more recent studies in Kazakhstan for horses^19^ and chickens^20^. Recognizing the importance of the different exposure pathways is already in the literature for ^3^H^21^, and many heavy metals, for example mercury^22^. There are also some arguments about the use of concentration ratios from Beresford and Willey^1^, however they remain to be seen as a powerful tool for predicting the effects of contamination. It also must be noted that available concentration ratio data, such as the IAEA Technical Report Series No 479^6^ might suffer from publication bias, and efforts are being made to add more data from non-temperate climates and identify other parameters significantly influencing the apparent concentration ratios^23^.

Giant African snails (*Lissachatina fulica*) are only native along the East African coast and to nearby islands^24^, however, have spread considerably as a result of deliberate or accidental transport by humans, moreover, are among the most invasive species^24,25^. They are also farmed and consumed in many countries, sold as pets and used for industrial applications like their slime in the pharmaceutical and cosmetics industries, as well as the application of their crushed shells as additives in ceramics, paint, animal feed, even in the construction and paper industries^26–28^. Accordingly, despite Giant African snails (*Lissachatina fulica*) being readily available in many countries, local legislation must be taken into account when planning research.

This paper describes the dynamics of ^137^Cs accumulation and its concentration ratio along with some stable elements in their body, shell, gastrointestinal tract and albumin gland following long-term ingestion (over 1, 20, 30, 40, 50 and 60 days) of contaminated forage and soil both separately and together.

## Results

### The activity concentration of radionuclides and concentration of stable elements in the sources of intake (contaminated forage, uncontaminated vegetables, and contaminated soil)

The results of the gamma-ray spectrometric analysis of forage, vegetables and soil are presented in Table 1.

**Table 1:** Activity concentration of radionuclides in soil, forage (grass meal) and vegetables.

Sample	Activity concentration, Bq kg^−1^ (DW)						
	^137^Cs	^241^Am	^152^Eu	^40^ K	^226^Ra	^232^Th	^90^Sr
Forage (grass meal)	376,000 ± 37,000	ND	ND	530 ± 50	ND	20 ± 2	14,000 ± 2000
Vegetable (control)	ND	ND	ND	1125 ± 100	ND	ND	ND
Soil (STS)	32,800 ± 3300	ND	ND	750 ± 75	35 ± 3	50 ± 5	19,000 ± 2000

ND, Not Detectable.

It was shown that the activity concentration of ^137^Cs in forage (~ 380 kBq kg^−1^, DW /dry weight) is one order of magnitude higher than in soil (~ 33 kBq kg^−1^, DW). The activity concentrations of ^241^Am and ^152^Eu in all samples were below the minimum detectable activity or equal to the background level.

The results following the elemental analysis of the forage (grass meal) and soil samples are presented in Table 2. In total, the activity concentrations of 16 elements were determined.

**Table 2:** Concentration of stable elements in the forage and soil, mg kg^-1^.

Element	Main source of radionuclides’ intake		Element	Main source of radionuclides’ intake	
	Forage (grass meal)	Soil (STS)		Forage (grass meal)	Soil (STS)
Na	450 ± 66	2500 ± 350	Co	0.09 ± 0.001	5.2 ± 0.7
Mg	1300 ± 200	5000 ± 600	Ni	4.8 ± 0.7	17 ± 2
Al	110 ± 17	22,500 ± 2900	Cu	7.6 ± 1.2	20 ± 2.6
K	13,000 ± 2000	5400 ± 670	Zn	81 ± 13	150 ± 20
Ca	5200 ± 770	10,000 ± 1300	Sr	34 ± 5	76 ± 10
Fe	190 ± 28	20,000 ± 2400	Cs	8.8 ± 1.4	4.6 ± 0.6
Cr	2.0 ± 0.3	27 ± 3	Pb	0.84 ± 0.13	35 ± 4
Mn	41 ± 6	520 ± 67	U	0.32 ± 0.01	99 ± 1

### Dynamics of ^137^Cs accumulation (Task A)

**Figure 1:**
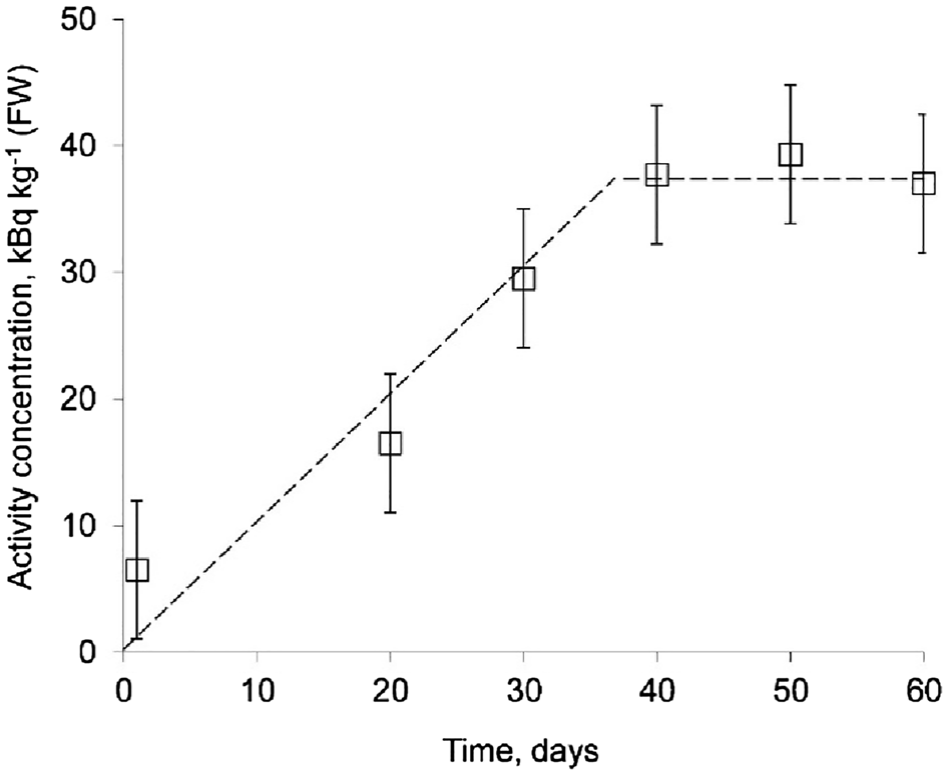
Dynamics of ^137^Cs uptake into the body of the snails during long-term ingestion of contaminated grass meal (number of snails − 30, including control group).

The activity concentrations of ^137^Cs in the bodies of snails fed with contaminated grass meal over a period of 1–60 days are presented in Fig. 1.

It is shown that following the prolonged intake of ^137^Cs, its activity concentration in the bodies of snails increases over the first 40 days of the experiment, after which a significant decrease in its rate of increase was observed. The activity concentration of ^137^Cs in the organs of snails reached almost 40 kBq kg^-1^, FW (fresh weight). The minimum (on Day 1) and maximum (on Day 60) ^137^Cs activity concentrations in their bodies differ by a factor of 7. The dashed line is a fit on the experimental data. Depending on species after an initial rapid uptake period, a slowed down uptake period and a dynamic equilibrium might be reached. The vertical bar is the measurement uncertainty of the pooled sample from 3 to 5 individuals belonging to the given treatment group from Supplementary Table 1. Compared to the trend reported for brown garden snails (*Cantareus aspersus,* formerly *Helix aspersa*) regarding the uptake kinetics of stable cesium by Pauget et al., radiocesium was bioavailable for the gastropods studied in this paper, reaching comparatively high activity concentrations of almost 40 kBq kg^−1^, FW^18^. Earlier uptake studies of caesium by gastropods were often over a shorter time period, e.g. Pauget et al. and Madoz-Escande & Simon used exposure times of 28 and 21 days, respectively^29^. There can be serious differences in the detoxification strategies between species^30^, so these results might not be directly comparable to freshwater or seawater snails. In addition, there might be differences between terrestrial species as well, as it was observed by Massadeth et al. for heavy metals and arsenic^31^.

### The distribution of ^137^Cs in the organs of the snails

**Figure 2:**
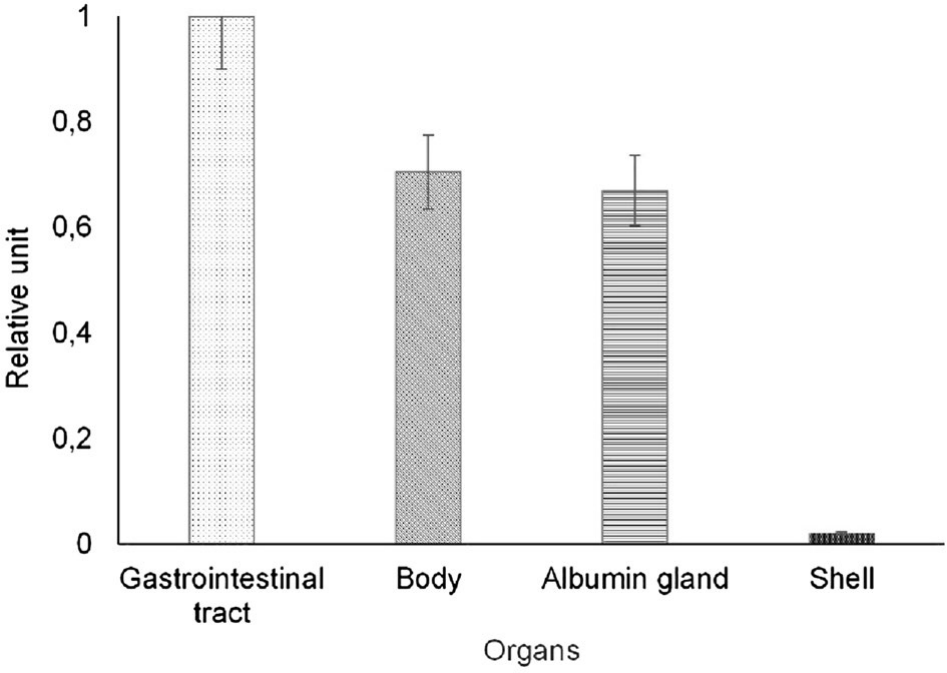
Distribution of ^137^Cs in the organs of snails (n = 28).

The distribution of ^137^Cs in the organs of the snails over a period during which its equilibrium state was more or less achieved, that is, 30 days, is shown in Fig. 2. The data are presented as a fraction of the maximum ^137^Cs activity concentration measured in the gastrointestinal tracts of these snails.

It can be seen from Fig. 2 that the distribution of ^137^Cs in the bodies of snails decreases as follows: gastrointestinal tract ˃ body = albumin gland ˃ shell. Madoz-Escande and Simon reported similar tendencies for *Helix aspersa maxima* with regard to its trophic pathway, however, when the direct deposition of radioactive aerosols is combined with a rain simulator, the ^137^Cs activity concentrations in the shell could be nearly as high as the levels near to the muscles or gastrointestinal tract^29^.

### Concentration ratio (C_R_) of ^137^Cs (Task B)

The calculated C_R_ of ^137^Cs for the individual organs of snails which were fed for 30 days are shown in Table 3. C_R_ is calculated as the ratio of the ^137^Cs activity concentrations in the organs (Bq kg^−1^, FW) to its activity concentration in soil (Bq kg^−1^, DW)^6^. In the case of group B1 where the snails were only fed with contaminated forage, C_R_ was calculated as the activity concentration in the organs (Bq kg^−1^, FW) divided by the activity concentration in the contaminated forage (Bq kg^−1^, DW).

**Table 3:** Concentration ratio of ^137^Cs calculated by activity concentration of ^137^Cs in the diet.

Organs	Group B1 A group of snails fed contaminated forage (C_R organs/forage_)	Group B2 A group of snails fed contaminated forage and soil (C_R organs/soil_)	Group B3 A group of snails fed contaminated soil (C_R organs/soil_)	TRS#479^6^ C_R (whole organism/soil)_, Mean min–max (n)
Body	0.057 ± 0.011	0.25 ± 0.05	0.004 ± 0.001	–
Shell	0.0011 ± 0.0003	0.012 ± 0.002	0.00024 ± 0.00005	–
Gastrointestinal tract	0.10 ± 0.02	0.43 ± 0.11	0.0067 ± 0.0013	–
Whole organism	–	–	–	0.04 0.021–0.065 (23)

It is shown that up to one order of magnitude is the difference between the C_R_ of groups B1 and B3. The difference in data between groups B2 and B3 was two orders of magnitude, meanwhile, only the C_R_ data of group B1 are comparable with data presented in IAEA handbook TRS 479^6^. This data show that the contribution of raising snails on contaminated soil is much lower than that of the forage contaminated with ^137^Cs on the concentrations observed in their bodies, so it could be argued that the C_R_ for group B2 to be calculated in relation to forage, and the uptake of radionuclides from soil to plants will play an important part in the concentrations developing in snails. This is in good agreement with Fritsch et al.^31^, who suggested the relative contribution of lettuce and soil to be 80 and 20%, respectively for *Cantareus aspersus*, however they also mention that the presence of earthworms might increase ^137^Cs uptake.

At the beginning of the experiment, it was expected that the activity concentration of ^137^Cs in groups B1 and B2 would be identical or at least not significantly different, since in both cases the snails were fed with the same contaminated forage. Nevertheless, the activity concentrations of ^137^Cs in the organs of snails from group B1 were twice as high as in group B2. It could be assumed that ^137^Cs uptake in the organs of group B2 was influenced by the stable Cs isotope or analogous elements contained within their feed and soil. However, the results of elemental analysis presented in Table 2 show that the content of stable Cs and K in the forage and soil as well as in their bodies did not differ. In fact, the concentration of the stable Cs isotope in snails from group B2 is two times less. Uptake from feed and from soil should be additional, but this was not observed in our study, unless the bedding changes feeding habits. Dietary metal exposure reportedly can reduce food consumption and inhibit growth in other snail species^32^. The feeding regime and the expected intake of the two groups was the same.

### Concentration ratio (C_R_) of heavy metals

Analysis of the elemental composition of the snails’ bodies in the three groups showed that the contents of trace elements (Na, Mg, Al, K, Ca, Fe, Cr, Mn, Co, Ni, Cu, Zn, Sr, Cs) is by and large comparable and do not depend on their feeding regime, with a few exceptions (Pu, U). The content of Pu and U in the snails from the three groups is worthwhile investigating since in those from groups B2 and B3 which were fed with contaminated soil, the concentration of Pu and U was significantly higher than in group B1; twice as high for Pb and 20–30 times as high for U. C_R_ of these two elements showed that Pb and U are intaken much more readily by snails from group B1 than by those from B2 or B3 as illustrated in Table 4.

**Table 4:** Concentration ratio of Pb and U in snails.

Element	*C*_*R*_		
	Group B1 A group of snails fed contaminated forage (C_R organs/forage_)	Group B2 A group of snails fed contaminated forage and soil (C_R organs/soil_)	Group B3 A group of snails fed contaminated soil (C_R organs/soil_)
Pb	0.083 ± 0.002	0.0043 ± 0.0008	0.0037 ± 0.001
U	0.13 ± 0.02	0.0086 ± 0.0020	0.014 ± 0.003

Regardless of the fact that the concentration ratios of Pb and U of snails fed with contaminated forage is one order of magnitude higher than those who in took soil, as seen in Table 5, the main contributing factor influencing the contamination of their bodies originates from the soil.

**Table 5:** Concentration ratio (C_R_) of ^137^Cs of snails with different weight.

Group #	Weight of snail, g	C_R (organs/forage)_	
		Body	Shell
C1	8.2 ± 1	(7.3 ± 1.5) × 10^–2^	(2.1 ± 0.4) × 10^–3^
C2*	18 ± 3	(5.7 ± 1.1) × 10^–2^	(1.1 ± 0.3) × 10^–3^
C3	71 ± 10	(8.1 ± 1.6) × 10^–2^	(8.2 ± 1.6) × 10^–4^
C4**	12 ± 1	(7.4 ± 1.5) × 10^–2^	n.a
Mean ± SD		(7.2 ± 1.1) × 10^–2^	(1.3 ± 0.2) × 10^–3^

*Data from group B1 as it was the same condition except for the weight of animals.

**Group C4 was fed Ca supplement in addition to contaminated forage.

Based on the obtained results regarding the Pb and U content in their bodies (see Supplement 2), it can be concluded that the soil is the main contributing factor that determines levels of contaminants in gastropods due to the much higher levels of contaminants in the soil than in the forage.

The observed Pb concentration ratios were lower than those reported by Gaso et al. for *Helix aspersa* in a semi-arid region of Mexico^9^, while Pauget et al. recorded similar results for U in *Cantareus aspersus*^18^. It must be noted that there might be differences between terrestrial snail species, as it was observed by Massadeth et al. for heavy metals and arsenic^31^ and soil physicochemical properties might also influence metal and metalloid uptake^33^.

### The influence of the age and weight of the snails on the variability of the transfer parameters of radionuclides (Task C)

The C_R_ of ^137^Cs that accumulated in the bodies and shells of snails of different ages and weights that were fed with contaminated forage for 30 days is shown in Table 5.

The table shows that regardless of their age or weight the C_R (forage)_ of ^137^Cs in their bodies is nearly identical, rendering its average value reliable. This is important when interpreting the data, since their growth could cause the real mass of the contaminants accumulated as a result of a dilution effect to be underestimated^18^.

## Discussion

This study provides up-to-date information on the transfer parameters of ^137^Cs and heavy metals into the organs of Giant African snails (*Lissachatina fulica*). The obtained results renders snails suitable as bioindicators and reference organisms when assessing ecosystems. It was found that forage was the main source of cesium contamination in the bodies of this species of snails, therefore, it is necessary to reconsider how the content of radionuclides in the bodies of wild animals, in particular gastropods, is assessed. Our findings, similar to Fritsch et al.^34^ suggest that the soil–plant–snail pathway has a higher influence on the developing activity concentrations in snails compared to the soil-snail route, but the latter is also not negligible. On the other hand, while the bioavailability of Pb and U in the snails that consumed forage was higher, due to the higher concentrations of contaminants in the soil, the latter source was the determining factor. In view of these results multiple environmental compartments and uptake pathways must be considered in biomonitoring applications and predictive dose modelling.

In addition, the absence of statistically significant differences in the transfer parameters of ^137^Cs in snails of different ages and weights will be useful to ensure environmental assessments are more accurate and comparable.

Some limitations should be considered when interpreting our results. There can be serious differences in the detoxification strategies between species^30^, so these results might not be directly comparable to freshwater or seawater snails. In addition, there might be differences between terrestrial species as well, as it was observed by Massadeth et al. for heavy metals and arsenic^30^. Furthermore, soil physicochemical properties might also influence metal and metalloid uptake^3^ and some sources suggest that even the presence of earthworms might influence the soil to snail uptake pathway^34^.

## Materials and methods

### Study outline

Terrestrial Giant African snails (*Lissachatina fulica*) 1 to 8 months in age with live weights of between 5 and 90 g were used to clarify if the aforementioned factors have any effect on the accumulation of Cs-137 and heavy metals. More than 60 specimens were divided into 13 groups, including two control groups, by randomly numbering them and using a random number generator. Furthermore, in Task C, groups based on their age and weight needed to be formed to test the effect of the accumulation of Cs-137. In vivo experiments were performed according to the guidelines of the ARRIVE and European Communities: Council Directive 86/609/EEC.

All the snails were kept in terraria, each individual occupied 0.007 m^3^ on average and each group consisted of 3–8 animals. The duration of the experiment varied from 1 to 60 days. All groups were fed once a day in the evening. Every day, residues of feed were removed and each terrarium rinsed with clean, uncontaminated water before the snails were put back into it and fed according to the feeding regime.

Their diet consisted mainly of fresh vegetables (*cucumis sativus, cucurbita pepo subsp.pepo*) and water. Radionuclides were added using contaminated forage and soil. For some selected groups, contaminated forage (grass meal—*Chamaenerion angustifolium, Cirsium arvense, Tanacetum vulgare, Calamagrostis arundinacea, Urtica dioica, Veronica spuria, Mentha interrupta, Rumex confertus, Geranium collinum, Sanguisorba officinalis, Delphinium dictyocarpum, *etc*.*) containing radionuclides was also fed to the snails, while for others, contaminated soil was poured into the terrarium as bedding.

Fresh vegetables were bought at the local market. The contaminated forage was harvested at the Semipalatinsk Test Site (STS). Forage was washed to remove dust, dried and ground in laboratory mills to form grass meal. Before feeding, 30 ± 3 g of finely cut cucumber or marrow and 4 g of contaminated grass meal were mixed until thoroughly homogenized. 5 to 12 g of mixed feed was placed in each terrarium, depending on the number and age of the snails. The following day before feeding, residues of forage were removed from the terraria.

The contaminated soil was collected from STS from a 1–2 m^2^ area at a depth of 0–5 cm and the soil cleared of large stones. The soil was mixed, homogenized and placed in a special storage container before a subsample was extracted from it for analysis.

The following three main tasks were investigated in this study: Task A—Determining the distribution and dynamics of the accumulation of radionuclides in the organs of snails by feeding with contaminated grass meal for up to 60 days; Task B—Determining the concentration ratio (C_R_) checking the effects of contaminated feed, raising on contaminated soil, and contaminated feed together with raising on contaminated soil for 30 days; Task C—Studying the influence of the age and weight of the snails against the transfer parameters (C_R_). The tasks were carried out simultaneously, with a joint control group. Group A3, B1 and C2 were the same, since that regime was overlapping between the three experiments.

Task A: In order to study the dynamics of the accumulation of radionuclides, the snails were divided into 6 subgroups and kept for different periods of time, namely 1, 20, 30, 40, 50 and 60 days, during which contaminated grass meal was added to their diet. In addition, two control groups were studied, the organs of which were examined both at the beginning and end of the experiment. Their weight at the beginning of the experiment was 17.6 ± 2.1 g and had increased on average by 2.5 ± 0.6 g by the end of the experiment.

Task B: In order to determine the transfer parameters of radionuclides and heavy metals (concentration ratio—C_R_) in the "forage-organism", "forage + soil-organism" and "soil-organism" food chains, the snails were divided into 3 groups and a feeding experiment carried out over 30 days. The snails were divided into 3 groups depending on the source of intake of radionuclides and heavy metals into their bodies as follows:

- Group B1 were fed with contaminated grass meal (group A3 renamed for this Task)
- Group B2 were fed with contaminated grass meal and contaminated soil was used as bedding
- Group B3 were fed with fresh vegetables and radioactively contaminated soil was used as bedding.

Task C: To assess the influence of the age and weight of the snails against the transfer parameters of radionuclides, they were divided into the following 4 subgroups:

- Group C1 consisted of snails with an initial live weight of 6.6 ± 0.4 g
- Group C2 (group A3 renamed for this Task) consisting of snails that initially weighed 17.6 ± 2.1 g
- Group C3 consisted of snails that initially weighed 74.7 ± 10.0 g
- Group C4 consisted of snails that initially weighed 8.4 ± 1.6 g.

All four groups were fed with contaminated grass meal mixed with clean vegetables prepared as described above for 30 days. Only the fourth group of snails (C4) was administered mineral feed supplements (shell rock and chalk) in addition to their daily diet. Data from this group will be used to study ^90^Sr transfer parameters. The weight of snails from group C1 increased by 1.5 ± 0.3 g, whereas the weight of group C3 decreased.

For the ethical handling of snails, a two-step method of euthanasia was used. In the first step, anesthesia is induced by immersion in 5% ethanol, followed by immersion in a euthanasia and tissue-preserving solution consisting of 70 to 95% ethanol recommended by Gilbertson and Wyatt^35^. In the second step, their organs were divided into four parts, that is, the shell, body (including its foot, head and mantle), internal organs and albumin gland. Preparation of the forage and soil as well as the radioanalytical procedure used have been described by Baigazinov et al.^19^.

To control the intake of radionuclides, samples of feed, soil and feces were taken throughout the experiment. Fecal sampling from all the groups was carried out on a daily basis during the first month of the experiment.

### Sample preparation and radioanalysis

The organs of the snails and fecal matter were measured when fresh, while the soil and forage were first dried. Fresh vegetable samples were bought before the feeding experiment commenced. Before gamma analysis, the samples were ground by a laboratory mill. The preparation process of the soil and grass meal samples adopted has been described by Mamyrbayeva^20^.

Gamma analyses were performed using a CANBERRA Ge BE3830 gamma radiation detector with a relative efficiency of 34% using Genie 2000 software. For the energy calibration of the spectrometer, a set of standard γ-sources manufactured by OSGI was used; while for geometric calibration, volumetric measurements of a given activity concentration standard (“OMACH” Rosatom) were used containing the radionuclides ^137^Cs, ^152^Eu and ^241^Am with an uncertainty of no more than 20%. The MDA for ^241^Am and ^137^Cs was 0.6 Bq kg^−1^. The methodology used for gamma analysis has previously been described^36^ and the laboratory given ISO 17025:2009 accreditation.

For elemental analysis, samples of soil, forage and snails’ organs underwent preliminary sample preparation by autoclave decomposition before being quantitatively transferred once cooled into a volumetric tube and brought to a volume of 15 cm^3^ with 1% nitric acid solution. The solution obtained in this way was diluted in a ratio of 1:100 and 1:10, respectively, and analyzed to determine the elemental content of interest.

The elemental content was determined by ICP-MS using a Thermo Scientific iCAP Q quadrupole mass spectrometer. To construct calibration curves, the multielement standard solutions KZ.03.02.00901-2010 and KZ.03.02.00902-2010 from the GSI RK register were used. Quality control of the measurements was carried out by measuring the calibration solution after every 10 samples. If the calibration result was unsatisfactory, that is, the calibration curve deviated by 8–10%, the instrument was recalibrated, taking into account the new background parameters.

The analysis was carried out according to the methodology described in ISO 17294-2׃2003 (E) “Water quality—Application of inductively coupled plasma mass spectrometry (ICP-MS)—Part 2: Definition of 62 elements” (state registration number 022/10505 of 12/27/05).

### Supplementary Information

Supplementary Tables.

## Data Availability

The data sets used and/or analyzed during the current study are available from the corresponding author on request.

## References

[1] Beresford NA, Willey N (2019). Moving radiation protection on from the limitations of empirical concentration ratios. J. Environ. Radioact..

[2] Howard BJ, Wells C, Barnett CL (2016). Improving the quantity, quality and transparency of data used to derive radionuclide transfer parameters for animal products. 1. Goat milk. J. Environ. Radioact..

[3] Howard BJ, Wells C, Barnett CL, Howard DC (2017). Improving the quantity, quality and transparency of data used to derive radionuclide transfer parameters for animal products. 2. Cow milk. J. Environ. Radioact..

[4] Sheppard SC (2011). Review of “Handbook of parameter values for the prediction of radionuclide transfer in terrestrial and freshwater environments”. J. Environ. Radioact..

[5] Yankovich TL, Beresford NA, Wood MD, Aono T, Andersson P, Barnett CL, Bennett P, Brown JE, Fesenko S, Fesenko J, Hosseini A, Howard BJ, Johansen MP, Phaneuf MM, Tagami K, Takata H, Twining JR, Uchida S (2010). Whole-body to tissue concentration ratios for use in biota dose assessments for animals. Radiat. Environ. Biophys..

[6] IAEA. Technical Reports Series no. 479 Handbook of Parameter Values for the Prediction of Radionuclide Transfer to Wildlife. In International Atomic Energy Agency, Vienna ISBN 978-92-0-100714-8 (2014).

[7] Beresford NA, Howard BJ (1991). The importance of soil adhered to vegetation as a source of radionuclides ingested by grazing animals. Sci. Total Environ..

[8] Beresford NA, Mayes RW, Cooker AI, Barnett CL, Howard BJ, Stuart Lamb C, Naylor GPL (2000). The importance of source-dependent bioavailability in determining the transfer of ingested radionuclides to ruminant-derived food products. Environ. Sci. Technol..

[9] Gaso I, Segovia N, Morton O (2002). In situ biological monitoring of radioactivity and metal pollution in terrestrial snails Helix aspersa from a semiarid ecosystem. Radioprotection.

[10] Gaso MI, Cervantes ML, Segovia N, Abascal F, Salazar S, Velazquez R, Mendoza R (1995). ^137^Cs and ^226^Ra determination in soil and land snails from a radioactive waste site. Sci. Total Environ..

[11] Dhiman V, Pant D (2021). Environmental biomonitoring by snails. Biomarkers.

[12] Hogan AC, van Dam RA, Houston MA, Harford AJ, Nou S (2010). Uranium exposure to the tropical duckweed *Lemna*
*aequinoctialis* and pulmonate snail *Amerianna*
*cumingi*: fate and toxicity. Arch. Environ. Contam. Toxicol..

[13] ICRP. Environmental Protection - the Concept and Use of Reference Animals and Plants. Annals of the ICRP. ICRP Publication 108. In *Annals of the ICRP*. ICRP Publication 108. (2008).

[14] Valentin J, Clarke RH, Holm LE (2003). A framework for assessing the impact of ionising radiation on non-human species. Ann. ICRP.

[15] Jandl J, Procházka H, Luks D (1991). The biological half-life of ^137^Cs in snails. J. Radioanal. Nucl. Chem. Let..

[16] Cœurdassier M, Gomot-de Vaufleury A, Lovy C, Badot PM (2002). Is the cadmium uptake from soil important in bioaccumulation and toxic effects for snails?. Ecotoxicol. Environ. Saf..

[17] De Vaufleury A, Cœurdassier M, Pandard P, Scheifler R, Lovy C, Crini N, Badot PM (2006). How terrestrial snails can be used in risk assessment of soils. Environ. Toxicol. Chem..

[18] Pauget B, Villeneuve A, Redon PO, Cuvier A, de Vaufleury A (2017). Assessment of the bioavailability and depuration of uranium, cesium and thorium in snails (*Cantareus*
*aspersus*) using kinetics models. J. Hazard. Mater..

[19] Baigazinov Z, Lukashenko SN, Panitsky VV, Kadyrova NZ, Karatayev SS, Mamyrbayeva SS, Baigazy S, Bazarbaeva, Kabdyrakova AB, Kunduzbaeva EE, Kenzhina LB, Zhadyranova AA, Hegedus M, Kovacs T (2020). The transfer of ^239+240^Pu, ^241^Am, ^137^Cs and ^90^Sr to the tissues of horses. J. Environ. Radioact..

[20] Mamyrbayeva AS, Baigazinov ZA, Lukashenko SN, Panitskiy AV, Karatayev SS, Shatrov AN, Baigazy SA, Bazarbayeva AB, Hegedűs M, Tóth-Bodrogi E, Kovács T (2020). The transfer of ^241^Am and ^137^Cs to the tissues of broilers’ organs. PLoS ONE.

[21] Baburajan A, Sudheendran V, Gaikwad RH, Ravi PM, Nayak RS, D’Souza SR, Karumakara N (2020). Tissue free water tritium (TFWT) and organically bound tritium (OBT) in marine eco system at Tarapur on the west coast of India. J. Radioanal. Nucl. Chem..

[22] FernandesAzevedo B, Barros Furieri L, Peçanha FM, Wiggers GA, FrizeraVassallo P, RonacherSimões M, Fiorim J, Rossi de Batista P, Fioresi M, Rossoni L, Stefanon I, Alonso MJ, Salaices M, ValentimVassallo D (2012). Toxic effects of mercury on the cardiovascular and central nervous systems. J. Biomed. Biotechnol..

[23] Doering C, Twining J, Rout S, Iurian A-R, Howard B (2021). A revised IAEA data compilation for estimating the soil to plant transfer of radionuclides in tropical environments. J. Environ. Radioact..

[24] Bernhard (2018). Hausdorf the giant African snail *Lissachatina*
*fulica* as potential index fossil for the Anthropocene. Anthropocene.

[25] Rasal V, Dhakad M, Khandal D (2022). Ecological invasion of the giant African snail *Lissachatina*
*fulica* (Bowdich, 1822) in a semi-arid forest of western India. Biodivers. Obs..

[26] Sunday E, Magu TO (2017). Determination of some metal contents in ashed and unashed snail shell powders. World News Nat. Sci..

[27] Noothuan N, Apitanyasai K, Panha S, Tassanakajon A (2021). Snail mucus from the mantle and foot of two land snails, *Lissachatina*
*fulica* and *Hemiplecta*
*distincta*, exhibits different protein profile and biological activity. BMC Res. Notes.

[28] Umarudin, Widyarti S, Waristo, Rahayu S (2022). Effect of Lissachatina fulica chitosan on the antioxidant and lipid profile of hypercholesterolemic male Wistar rats. J. Pharm. Pharmacogn. Res..

[29] Madoz-Escande C, Simon O (2006). Contamination of terrestrial gastropods, Helix aspersa maxima, with ^137^Cs, ^85^Sr, ^133^Ba and ^123^mTe by direct, trophic and combined pathways. J. Environ. Radioact..

[30] Dvorak M, Schnegg R, Niederwanger M, Pedrini-Martha V, Ladurner P, Lindner H, Kremser L, Lackner R, Dallinger R (2019). cadmium pathways in snails follow a complementary strategy between metallothionein detoxification and auxiliary inactivation by phytochelatins. Int. J. Mol. Sci..

[31] Massadeh AM, Alomary AA, Mir S, Momani FA, Haddad HI, Haddad YA (2016). Analysis of Zn, Cd, As, Cu, Pb, and Fe in snails as bioindicators and soil samples near traffic road by ICP-OES. Environ. Sci. Pollut. Res..

[32] El-Gendy KS, Radwan MA, Gad AF (2011). Feeding and growth responses of the snail theba pisana to dietary metal exposure. Arch. Environ. Contam. Toxicol..

[33] Baroudi F, Al ALam J, Fajloun Z, Millet M (2020). Snail as sentinel organism for monitoring the environmental pollution; a review. Ecol. Indic..

[34] Fritsch C, Scheifler R, Beaugelin-Seiller K, Hubert P, Coeurdassier M, de Vaufleury A, Badot PM (2008). Biotic interactions modify the transfer of cesium-137 in a soil-earthworm-plant-snail food web. Environ. Toxicol. Chem..

[35] Gilbertson CR, Wyatt JD (2016). Evaluation of euthanasia techniques for an invertebrate species, land snails (*Succinea*
*putris*). J. Am. Assoc. Lab. Anim. Sci..

[36] Measurement technique on a gamma spectrometer. The activity of radionuclides in bulk samples. MI 2143-91: MI 5.06.001.98 RK. Almaty; 1998. p.18 (in Russian) (1988).

