# Supplementary material for: The transfer of 137Cs and heavy metals to tissues within the organs of snails
Source: Sci Rep. 2023 Sep 21;13:15690. doi: 10.1038/s41598-023-42580-6 (PMC10514058; doi:10.1038/s41598-023-42580-6)
Supplement: Supplementary file 1 — Supplementary Tables. [file 41598_2023_42580_MOESM1_ESM.docx]

Supplementary 1

Activity concentration of radionuclides in snails’ organs

| Group # | Duration, days | Number of animals | Weight of snails, mean±SD | | Main source of radionuclides intake | Organs | Activity concentration, Bq kg^-1^ (FW) | | | | | |
| --- | --- | --- | --- | --- | --- | --- | --- | --- | --- | --- | --- | --- |
|  |  |  | before the experiment | at the end of experiment |  |  | ^137^Cs | ^241^Am | ^152^Eu | ^40^K | ^226^Ra | ^232^Th |
| Task A | | | | | | | | | | | | |
| Control  A0 | 1 | 6 | - | 6±1 | - | Body | BDA | BDA | BDA | BDA | 30±3 | BDA |
| A1 | 1 | 5 | - | 14.2 | Forage | Body | 6500±650 | BDA | BDA | BDA | 30±3 | BDA |
|  |  |  |  |  |  | Shell | 213±20 | BDA | BDA | BDA | BDA | BDA |
|  |  |  |  |  |  | Gastrointestinal tract | 11500±1150 | BDA | BDA | BDA | BDA | BDA |
| A2 | 20 | 5 | 15.1 | 15.1 | Forage | Body | 16500±1650 | BDA | BDA | BDA | BDA | BDA |
| A3 | 30 | 5 | 17.6±2.1 | 18.2±1.9 | Forage | Body | 21600±2100 | BDA | BDA | BDA | 30±3 | 13±1 |
| A4 | 40 | 3 | - | 18.0 | Forage | Body | 37700±3800 | BDA | BDA | BDA | BDA | BDA |
| A5 | 50 | 3 | - | 15.5 | Forage | Body | 39300±4000 | BDA | BDA | BDA | BDA | BDA |
| A6 | 60 | 3 | - | 15.3 | Forage | Body | 36150±4000 | BDA | BDA | BDA | BDA | BDA |
| Task B | | | | | | | | | | | | |
| Control  B0 | 30 | 3 | 53.8±2 | 56±2 | - | Body | BDA | BDA | BDA | BDA | 19±2 | BDA |
| B1 | 30 | 5 | 17.6±2.1 | 18.2± 1.9 | Forage | Body | 21600±2100 | BDA | BDA | BDA | 30±3 | 13±1 |
|  |  |  |  |  |  | Shell | 420±40 | BDA | BDA | BDA | BDA | BDA |
|  |  |  |  |  |  | Gastrointestinal tract | 38000±3800 | BDA | BDA | BDA | BDA | BDA |
| B2 | 30 | 5 | 18.9 | 21.5 | Soil and forage | Body | 8100±800 | BDA | BDA | BDA | 20±2 | BDA |
|  |  |  |  |  |  | Shell | 402±40 | BDA | BDA | BDA | BDA | BDA |
|  |  |  |  |  |  | Gastrointestinal tract | 14053±1405 | BDA | BDA | BDA | BDA | BDA |
|  |  |  |  |  |  | Albumin gland | 10078±1000 | BDA | BDA | BDA | BDA | BDA |
| B3 | 30 | 5 | 19.1 | 22.1 | Soil | Body | 130±13 | BDA | BDA | BDA | 13±1 | BDA |
|  |  |  |  |  |  | Shell | <8 | BDA | BDA | BDA | BDA | BDA |
|  |  |  |  |  |  | Gastrointestinal tract | 220±20 | BDA | BDA | BDA | BDA | BDA |
| Task C | | | | | | | | | | | | |
| C1 | 30 | 7 | 6.6 | 8.2 | Forage | Body | 27630±2760 | BDA | BDA | BDA | BDA | BDA |
|  |  |  |  |  |  | Shell | 800±80 | BDA | BDA | BDA | BDA | BDA |
|  |  |  |  |  |  | Gastrointestinal tract | 39000±3900 | BDA | BDA | BDA | BDA | BDA |
| C2* | 30 | 5 | 17.6±2.1 | 18.2± 1.9 | Forage | Body | 21600±2100 | BDA | BDA | BDA | 30±3 | 13±1 |
|  |  |  |  |  |  | Shell | 420±40 | BDA | BDA | BDA | BDA | BDA |
|  |  |  |  |  |  | Gastrointestinal tract | 38000±3800 | BDA | BDA | BDA | BDA | BDA |
| C3 | 30 | 3 | 74.7 | 70.5 | Forage | Body | 30400±3040 | BDA | BDA | BDA | BDA | BDA |
|  |  |  |  |  |  | Shell | 300±30 | BDA | BDA | BDA | BDA | BDA |
|  |  |  |  |  |  | Gastrointestinal tract | 25000±2500 | BDA | BDA | BDA | BDA | BDA |
|  |  |  |  |  |  | Albumin gland | 15600±1500 | BDA | BDA | BDA | BDA | BDA |
| C4 | 30 | 7 | 8.4 | 12.0 | Forage + Ca | Body | 28000±2800 | BDA | BDA | BDA | BDA | BDA |
| Note: * - data from group B1 as it was the same condition except for the weight of animals | | | | | | | | | | | | |

Supplementary 2

Concentration of stable elements in the body of snails, forage and soil, mg kg^-1^

| Element | Body of snails | | |
| --- | --- | --- | --- |
|  | Group B1.  A group of snails fed contaminated  forage, raised on clean soil | Group B2.  A group of snails fed contaminated  forage, raised on contaminated soil | Group B3.  A group of snails fed fresh  forage, raised on contaminated  soil |
| Na | 2100±300 | 2000±300 | 2000±280 |
| Mg | 3100±440 | 1600±230 | 2200±320 |
| Al | 3.8±0.6 | 5.4±0.8 | 5.5±1 |
| K | 4000±580 | 4100±600 | 4800±700 |
| Ca | 7100±1000 | 5500±800 | 6500±900 |
| Fe | 47±7 | 48±7 | 43±6 |
| Cr | 0.7±0.1 | n.a. | 0.7±0.1 |
| Mn | 5.1±0.8 | 4.6±0.7 | 28±4 |
| Co | 0.031±0.005 | 0.022±0.003 | 0.021±0.003 |
| Ni | 0.80±0.12 | 0.72±0.11 | 0.74±0.12 |
| Cu | 24±4 | 22±3.3 | 21±4 |
| Zn | 61±10 | 43±7 | 66±10 |
| Sr | 210±30 | 150±23 | 230±40 |
| Cs | 3.4±0.5 | 1.2±0.2 | n.a. |
| Pb | 0.07±0.01 | 0.15±0.02 | 0.13±0.02 |
| U | 0.04±0.01 | 0.85±0.1 | 1.4±0.2 |

Note: n.a. – not available
